# Increased meso-striatal connectivity mediates trait impulsivity in *FTO* variant carriers

**DOI:** 10.3389/fendo.2023.1130203

**Published:** 2023-05-08

**Authors:** Sharmili Edwin Thanarajah, Ruth Hanssen, Corina Melzer, Marc Tittgemeyer

**Affiliations:** ^1^ Max Planck Institute for Metabolism Research, Cologne, Germany; ^2^ Department for Psychiatry, Psychosomatic Medicine and Psychotherapy, University Hospital Frankfurt, Goethe University, Frankfurt am Main, Germany; ^3^ Faculty of Medicine and University Hospital Cologne, Policlinic for Endocrinology, Diabetes and Preventive Medicine (PEPD), University of Cologne, Cologne, Germany; ^4^ Cluster of Excellence in Cellular Stress Responses in Aging-associated Diseases (CECAD), Cologne, Germany

**Keywords:** FTO, dopamine, connectivity, impulsivity, structure, nucleus accumbens

## Abstract

**Objective:**

While variations in the first intron of the *fat mass and obesity-associated gene* (*FTO*, rs9939609 T/A variant) have long been identified as a major contributor to polygenic obesity, the mechanisms underlying weight gain in risk allele carriers still remain elusive. On a behavioral level, *FTO* variants have been robustly linked to trait impulsivity. The regulation of dopaminergic signaling in the meso-striatal neurocircuitry by these *FTO* variants might represent one mechanism for this behavioral alteration. Notably, recent evidence indicates that variants of *FTO* also modulate several genes involved in cell proliferation and neuronal development. Hence, FTO polymorphisms might establish a predisposition to heightened trait impulsivity during neurodevelopment by altering structural meso-striatal connectivity. We here explored whether the greater impulsivity of *FTO* variant carriers was mediated by structural differences in the connectivity between the dopaminergic midbrain and the ventral striatum.

**Methods:**

Eighty-seven healthy normal-weight volunteers participated in the study; 42 FTO risk allele carriers (rs9939609 T/A variant, *FTO* ^+^ group: AT, AA) and 39 non-carriers (*FTO* ^−^ group: TT) were matched for age, sex and body mass index (BMI). Trait impulsivity was assessed via the Barratt Impulsiveness Scale (BIS-11) and structural connectivity between the ventral tegmental area/substantia nigra (VTA/SN) and the nucleus accumbens (NAc) was measured via diffusion weighted MRI and probabilistic tractography.

**Results:**

We found that *FTO* risk allele carriers compared to non-carriers, demonstrated greater motor impulsivity (*p* = 0.04) and increased structural connectivity between VTA/SN and the NAc (p< 0.05). Increased connectivity partially mediated the effect of FTO genetic status on motor impulsivity.

**Conclusion:**

We report altered structural connectivity as one mechanism by which *FTO* variants contribute to increased impulsivity, indicating that *FTO* variants may exert their effect on obesity-promoting behavioral traits at least partially through neuroplastic alterations in humans.

## Highlights

- Human healthy-weight carriers of the fat mass and obesity-associated (*FTO*) risk allele variant rs9939609 show enhanced trait impulsivity
- *FTO* risk allele carriers also demonstrate increased structural meso-striatal connectivity
- The greater impulsivity related to variants of *FTO* is partially mediated by the increased structural connectivity between dopaminergic midbrain and nucleus accumbens suggesting a mechanism by which genetic predisposition might affect this behavioral trait in humans

## Introduction

Obesity results from an interplay between obesogenic environmental factors and individual (genetic) susceptibility (1). In 2007, variations in the first intron of the fat mass and obesity-associated gene (FTO) were identified to be associated with increased body mass (2). This association has since been replicated in several populations of children and adults across both genders, providing now-established evidence that variations in the FTO gene contribute to polygenic obesity (3–6). However, the underlying molecular mechanisms are entwined affecting multiple organs and processes from thermogenesis in brown adipose tissue and lipid storage (7) to encoding of adaptive behavior in the brain (8). Furthermore, the direct and indirect molecular mechanisms of FTO are particularly complicated to disentangle, as FTO polymorphisms are not only of functional relevance for behavioral encoding in the adult brain but also affect a multitude of signaling pathways during early development causing structural brain changes and hence potentially the basis for hard-wired predispositions for behavioral alterations.

The (neuro)developmental effects of FTO are based on its role as a demethylase. Specifically, FTO modulates the expression of several genes involved in cell proliferation, migration, and neuronal development (9–14). Loss of *FTO* leads to the altered expression of several key components of the brain-derived neurotrophic factor pathway [BDNF, 13], which has been repeatedly linked to polygenic and mono-genetic obesity forms via several molecular mechanisms (15–18). Loss of *FTO* also reduces the pool of adult neural stem cells and leads to decreased brain size, deficient neurogenesis (10, 13, 19)

and hypomyelination (20). Additionally, evidence from animal studies also indicates an indirect role of *FTO* polymorphism in neuronal plasticity promoting obesity by regulating genes or enhancers involved in neurogenesis relevant for body weight homeostasis (21–26) without affecting FTO expression (26). Hence, *FTO* polymorphism might - directly and indirectly - affect obesity development via neurodevelopmental pathways.

In addition to its effects on neuronal plasticity, FTO polymorphisms also affect the neural encoding of behavior in adulthood. Due to the regulatory role of *FTO* polymorphisms in FTO function as demethylase, intronic variants affect the modification of specific mRNAs of critical components of D2/3R-signaling (8) and consequently D2/D3 receptor-mediated auto-inhibition. Thus, *FTO* regulates the activity of DA neurons in the basic reward circuit of meso-striatal regions altering reward sensitivity (8). Evidence from human studies links risk-variants to alterations in multiple reward-related behaviors such as increased preference for high-caloric food (27, 28), heightened food craving (29), differences in food-cue reactivity (30), impaired reward learning (31) and alterations in impulse control (32).

Among the behavioral traits affected by *FTO* and encoded by the DA reward circuitry, impulsivity has been consistently associated with *FTO* polymorphism (32–34) and increased risk for future weight gain and overeating (35–38). According to the conceptualization of trait impulsivity by the Barett Impulsiveness Scale (BIS), particularly attentional (impaired ability to focus or concentrate) and motor (acting without thinking) impulsivity interact to promote weight gain (39). As the regulation of motor impulsivity predominantly depends on intact DA functioning in the ventral striatum (40) and given the regulatory role of FTO in D2-receptors (D2R) signaling in striatal regions, *FTO* might affect obesity development by regulating dopaminergic encoding of motor impulsivity in the midbrain. Indeed, this role is supported by our previous finding that *FTO* modulates increased functional coupling in meso-striatal regions —specifically ventral tegmental area/substantia nigra (VTA/SN) and the nucleus accumbens (NAc) as well as further downstream targets such as the medial prefrontal cortex (31). It is, however, unclear if FTO polymorphisms contribute to these alterations in the dopaminergic encoding of reward-related behavior via their neurodevelopmental effect on structural connectivity in the meso-striatal system. We, therefore, studied the structural connectivity of projections from the midbrain (VTA/SN) to the ventral striatum (NAc) and its contribution to the impulsive phenotype of FTO risk allele carriers.

## Methods

### Participants and study design

A cohort of 589 healthy, young Caucasians enrolled in the University of Cologne were recruited and genotyped for the rs9939609 T/A variant of the FTO gene (hereafter: *FTO* ^–^ group (TT) and FTO^+^ group (AT, AA)) as previously described (30, 31).

Participants were invited for further partially overlapping sub-studies. Eighty-seven participants were included in this sub-study, in which all participants underwent an impulsivity assessment using the Barrattt Impulsivity Scale-11 among other questionnaires (Becks Depression Inventory (BDI-2), Sensitivity to Punishment and Reward Questionnaire (SPSRQ), Alcohol Use Disorders Identification Test (AUDIT)), a buccal swab for DNA isolation and genotyping, a blood draw to assess the metabolic status and MRI imaging. The assessment of the Barrattt Impulsivity Scale and DNA isolation from the buccal swabs were performed as part of a previously published study (31). Inclusion criteria for the analysis performed in this study comprised: fully answered BIS-11 questionnaire and complete MRI imaging, no known diseases, fasting glucose and insulin within normal range, BDI-2 within normal range. 6 participants had to be excluded from further analyses due to unanswered BIS-11 questionnaire (5) and suspected diabetes mellitus (1) as indicated by a Homeostasis Model Assessment of Insulin Resistance (HOMA-IR, (41)) >5. In total, eighty-one participants were included in the final analysis (**Table 1**), 39 non-carriers (FTO^–^; 24.8 ± 3.5 years, 22.6 ± 2.6 kg/m^2^) and 42 risk allele carriers (FTO^+^; 25.7 ± 4.4 years; 23.2 ± 3.2 kg/m^2^).

**Table 1:** Participants’ characteristics.

	*FTO* ^+^	*FTO* ^–^		
*N*	42	39		
Gender (male/female)	18/24	16/23	*X^2^* (1) = 0.03	*p* = 0.87
Age [years]	25.0 ± 0.6	24.8 ± 0.6	*t*(71.9) = -0.26	*p* = 1
BMI [kg/m^2^]	23.1 ± 0.5	22.6 ± 0.4	*t*(78,1) = -0.81	*p* = 0.42
Waist to Hip ratio	0.81 ± 0.02	0.85 ± 0.01	*t(63.5) = 1.35*	*p = 0.20*
Insulin [mU/l]	5.3 ± 0.6	6.0 ± 0.6	*t(63.0) = 0.78*	*p = 0.44*
Glucose [mg/dl]	84.5 ± 1.3	83.3 ± 1.2	*t(64.0) =- 0.71*	*p = 0.48*
HOMA-IR	1.1 ± 0.1	1.2 ± 0.1	*t(78.4) =-0.41*	*p = 0.68*
Cholesterol[mg/dl]	181.5 ± 5.0	175.1 ± 6.4	*t(58.7) =- 0.79*	*p = 0.43*
Triglycerides [mg/dl]	95.2 ± 9.4	94.4 ± 10.9	*t(61.3) =- 0.05*	*p = 0.96*
BDI-2	3.2 ± 0.6	3.7 ± 0.7	*t(76.5) = 0.48*	*p = 0.63*
FEV Scale 1: Cognitive control of food intake behavior	5.9± 0.8	6.9± 0.7	*t(76.9) = 0.91*	*p = 0.37*
FEV Scale 2: Disturbability of food intake behavior	5.3± 0.4	5.2± 0.4	*t(77.3) = -0.15*	*p = 0.88*
FEV Sacel 3: Experience of hunger feelings	5.4± 0.5	4.5± 0.4	*t(75.2) = -1.41*	*p = 0.16*
SPSRQ: Sensitivity to Punishment	7.7± 0.7	9.5± 0.6	*t(78.7) = 1.88*	*p = 0.06*
SPSRQ: Sensitivity to Reward	11.2± 0.6	10.7± 0.6	*t(79.0) = -0.55*	*p = 0.58*
AUDIT	6.4± 0.6	6.1± 0.8	*t(74.8) = -0.30*	*p = 0.77*

Mean with standard error of the mean are depictured; p-values of t-test are not corrected for multiple comparisons. X2, chi-square; FTO−: TT; FTO+: AT; AA; WtHr, Waist to hip ratio; HOMA-IR, Homeostasis Model Assessment of Insulin Resistance; BDI, Becks Depression Inventary; FEV, German version of Three-Factor-Eating Questionnaire (Fragebogen zum Essverhalten). SPSRQ, Sensitivity to Punishment and Reward Questionnaire; AUDIT, Alcohol Use Disorders Identification Test.

All participants gave written informed consent to participate in the experiment, which had been approved by the local ethics committee of the Medical Faculty of the University of Cologne (Cologne, Germany, 10-226).

### Assessment of impulsivity (BIS-11)

Impulsivity in participants was assessed by the German version of the BIS-11 questionnaire (42). The BIS-11 is the most often used self-report questionnaire to assess trait impulsivity (43). It consists of 30 items and is divided into 6 first-order (Attention, Motor, Self-control, Perseverance, Cognitive Instability, Cognitive Complexity) and 3 second-order factors (Attentional impulsiveness, Motor impulsiveness, and Non-planning impulsiveness). Each item is measured on a 4-point ‘Likert Scale’ ranging from “rarely/never” to “almost always/always”. The total score is calculated by summing all the item ratings, of which 8 cover attentional impulsiveness, 11 motor impulsiveness, and 11 non-planning impulsiveness, with a possible total range from 30 to 120.

### DNA isolation and SNP-genotyping

Isolation of DNA from buccal swabs was performed using the QIAamp DNA Blood Mini Kit (# 51106, Qiagen GmbH, Hilden, Germany) according to the manufacturer’s instructions. The concentration and quality of the DNA were determined with a ND-1000 UV/Vis-Spectrophotometer (Peqlab GmbH, Erlangen, Germany). SNP genotyping for rs9939609 (*FTO*) was performed with 20 ng of DNA in triplicates using allelic discrimination assays (TaqMan SNP Genotyping Assays, Applied Biosystems by Life Technology GmbH, Darmstadt, Germany). The genotyping PCR was carried out on a 7900HT Fast Real-Time PCR System (Applied Biosystems) and the resulting fluorescence data were analyzed with Sequence Detection Software (SDS) 2.3 (Applied Biosystems) as already previously described for this cohort of patients.

### Blood draw

Blood was drawn after an overnight fasting period of at least 8 hours to determine insulin, glucose, cholesterol and triglyceride values to compare the metabolic state of the participants. Insulin resistance was calculated according to the homeostatic model of insulin resistance (HOMA-IR) (41).

### MRI acquisition

Imaging was performed on a 3 Tesla MRI scanner (Siemens TIM Trio, Erlangen, Germany). First, for each subject structural MRI data sets were acquired in a 12-channel array head coil and a maximum gradient strength 40 mT/m with a whole-brain field of view (T1-weighted: MDEFT3D; TR = 1930 ms, TE = 5.8 ms, resolution 1.0 × 1.0 × 1.25 mm, flip angle 18°, sagittal; T2-weighted: RARE; TR = 3200 ms, TE = 458 ms, 176 sagittal slices, resolution 1.0 × 1.0 × 1.0 mm^3^). Subsequently diffusion-weighted magnetic resonance imaging (dMRI) was conducted using spin echo planar imaging [SE-EPI; TR = 12,000 ms, TE = 100 ms, resolution 1.7 × 1.7 × 1.7 mm^3^, flip angle: 90°, Field of View (FoV): 220 × 220 × 122 mm^3^, bandwidth: 1345 Hz/pixel, orientation: axial, data matrix: 128 × 128 × 72, PAT factor 2, partial Fourier 6/8] with double-spin echo preparation (44). Diffusion-weighting was isotropically distributed along 60 diffusion-weighted directions (b value = 1000 s/mm^2^). Additionally, in each subject, seven data sets with no diffusion weighting (B0) were acquired initially and interleaved after each block of 10 diffusion-weighted images as anatomical reference for motion correction. To optimize the signal-to-noise ratio, when available scans from three assessments were averaged.

### Masks

**Figure 1:**
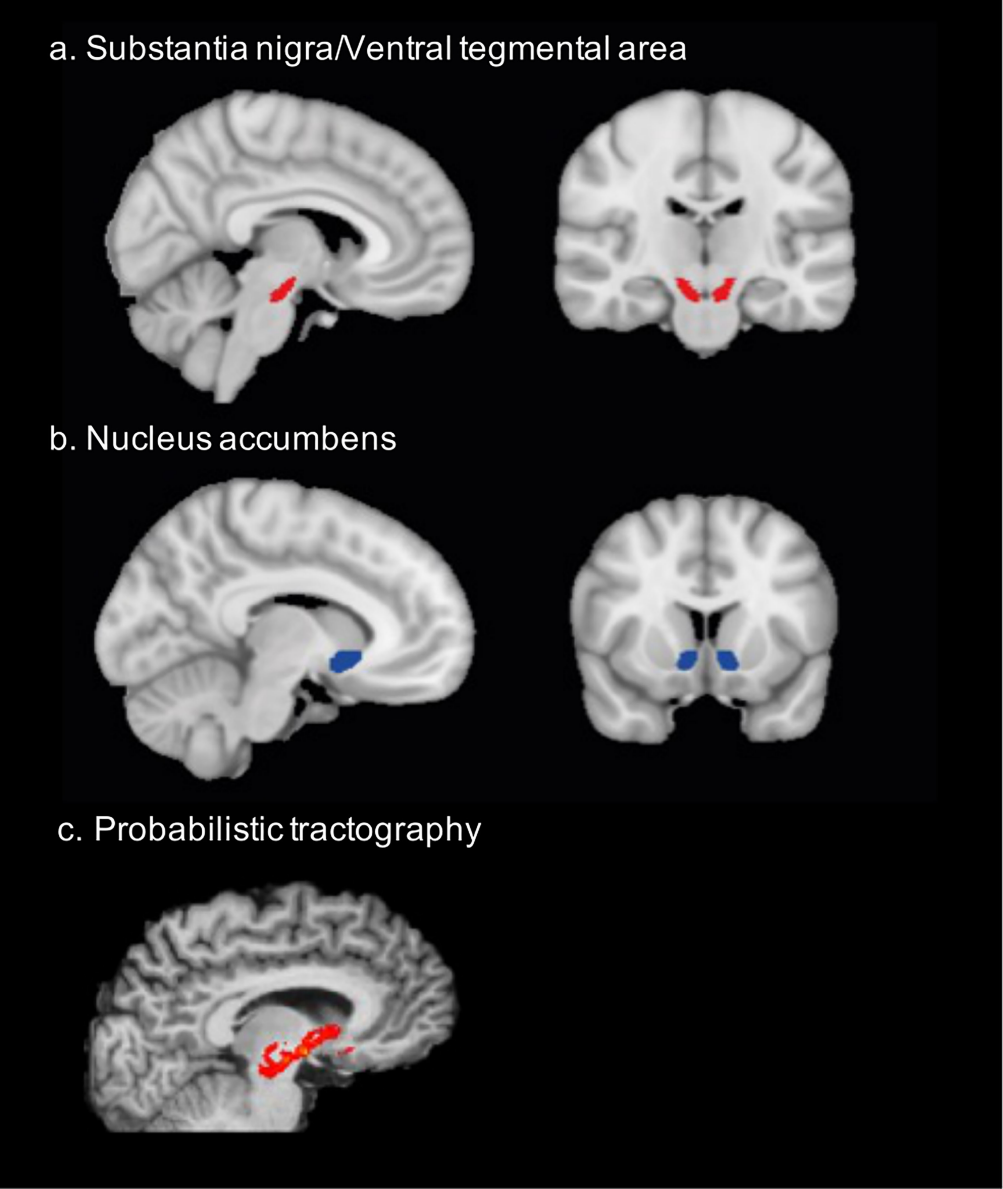
Structural connectivity was assessed between **(A)** the ventral tegmental area/substantia nigra (x=96, y= 106, z=53) and **(B)** the nucleus accumbens (x=99, y=138, z=62). The mask location comprising the midbrain (VTA/SN) was chosen upon comparison across standard anatomical atlases as detailed in Pelzer et al. (47). The NAc mask was retrieved from the Harvard-Oxford subcortical atlas. **(C)** probabilistic tractography was performed between these masks to assess structural connectivity. This is the example of one participant.

To create seed masks informing the tractography approach, we used anatomical constraints to outline the regions of VTA/SN, NAc, and caudate nucleus. Specifically, the mask location comprising the midbrain (VTA/SN) was chosen upon comparison across standard anatomical atlases (45, 46) as detailed in Pelzer et al. (47). For the midbrain mask, the following structures marked the border: ventromedially the red nucleus, latero-dorsally the subthalamic nucleus, and anteriorly the cerebral peduncles. The SN/VTA mask was labeled on an average T2-image in standard space in axial plane, and subsequently checked in coronal and sagittal plane. The masks for NAc and the caudate were retrieved from the Harvard-Oxford subcortical atlas (48, 49). Initially labeled in MNI-152-1mm space, the masks were afterwards transformed to individual diffusion space using the FSL’s applywarp function (50) and visually checked for each participant (**Figure 1**).

### MRI data analysis

Preprocessing and data analysis were performed using tools from the FMRIB software library (FSL, version 5.0.4, https://www.fmrib.ox.ac.uk/fsl): First, we applied FSL’s brain extraction tool (BET) to T1-weighted (T1w) images and applied the resulting brain mask to T2-weighted (T2w) images, that had been aligned to T1-weighted images using rigid body alignment. Diffusion-weighted images were corrected for head motion using affine registration [FLIRT; 51] while using the first acquired B0 as a reference volume. Based on this transformation, the original *b*-vectors were corrected for the rotational components. To further enhance the reliability of the upcoming tractography algorithm, the preprocessed diffusion-weighted data were interpolated to 1mm (51, 52). T1-, T2- weighted images were then linearly co-registered using FSL’s FLIRT tool (53) to the MNI-152 1mm isotropic brain (12 DOF-affine registration). Diffusion-weighted images were co-registered to standard space using non-linear registration (FNIRT). The anatomical masks were nonlinearly transformed from standard space to diffusion space by applying the transformation matrices acquired with FSL’s FNIRT function (54). FSL’s BedpostX program was applied to model diffusion parameters for crossing fibers in native space. Afterwards, probabilistic tractography was performed in diffusion space using FSL’s Probtrackx2 (53, 55, 56). Probabilistic tractography is leveraged to reconstruct white matter structural connectivity in diffusion data. Starting from a voxel in the seed mask the tracking is performed in a stepwise manner from a distribution of possible directions. The distributions of principal diffusion directions calculated through BedpostX were used at each voxel to compute a streamline through these local samples and generate a probabilistic streamline. By taking many such samples, the FMRIB’s diffusion toolbox (FDT) is able to build upon the posterior distribution on the streamline location or the connectivity distribution (55). For each subject and seed region, the following parameters were chosen: The number of samples was 5000 for each seed voxel with a curvature threshold of 0.2, a maximum number of steps of 2000 with a step length of 0.5 mm. A path distribution function was added in order to correct for differences in distance between seed and target regions. The tractography was performed twice between seed and target in either direction. The ventricles were included as exclusion masks.

### Connectivity measure

Structural connectivity between the SN/VTA and the NAc was analyzed. In order to test for construct validity, we also tested for differences in the structural connectivity between the DA midbrain and caudate, which is more implicated in attentional impulsivity than motor impulsivity and hence served as a validator for the specificity of the expected alterations in VTA/SN-NAc connectivity. The connectivity measure was defined as described in Stephan et al. (57). From the seed region (*S*) 5000 samples were initiated, and the proportion of the samples (K_S_) that intersected with target (*T*) defined the connectivity between these areas. Hence, this quantity reflects a relative connection density yielding numbers between 0 and 1 and does not inform about the absolute number of connections between these areas (58). It is important to note that this measure differs depending on whether tractography is sourced from *S* or *T*. Therefore, we performed the tractography in both directions and normalized by the size of the source region (N). Hence the relative connectivity density (φ_ST_) was defined by the following formula:


φST =12 × (KSNS×5000+KTNT×5000)

For statistical analyses this connectivity measure was considered to assess connectivity between the VTA/SN and NAc. This connectivity measure was also used to assess connectivity between the VTA/SN and the caudate in order to demonstrate the specificity of the effect of *FTO* polymorphism on structural connectivity between VTA/SN and NAc. For both VTA/SN-NAc and VTA/SN-caudate, connectivity measures were analyzed averaged across hemispheres.

### Statistical analysis

Data was analyzed using RStudio [version 1.4.1717; (59)] and R [version 4.0.0; (60)] using the functions of R’s ‘Stats’ package (version 3.6.2) if not stated otherwise. Frequency distribution was tested with the Shapiro-Wilk-Test. For the connectivity data the Shapiro-Wilk-Test resulted in a deviation (*p*< 0.001) from the normal distribution. Due to this deviation from the normal distribution, we performed a logarithmic transformation of the connectivity values before further parametric testing. For further statistical assessment, linear regression models were performed using the “*lm*”-function in R. First, a linear regression model was used to assess differences in the total score of the BIS-11 scale between both groups (*FTO* ^+^ vs *FTO* ^–^, **Table S1**). To test for differences in the second-order factors of the BIS-11 (attentional impulsiveness, motor impulsiveness and non-planning impulsiveness) between the *FTO* ^+^ and the *FTO* ^–^ group we also applied linear regression models (**Table S2**). We controlled for multiple comparisons using Bonferroni correction. Then we analyzed differences in (log) connectivity between the two groups (**Table S3**) and tested for the effect of FTO risk allele carrier status and (log) connectivity on motor impulsiveness in a combined model as required for the following mediation analysis (**Table S4**) using linear regression models respectively. Last, a mediation analysis was performed to test if (log) connectivity mediates the effect of risk allele carrier status on motor impulsiveness using R’s ‘*Mediation*’ package [version 4.5.0; (61); **Table S5**]. Effect sizes were calculated using R’s package ‘*Effectsiz*e’ [version 0.4.5; (62)]. Statistical significance was reported at a level of *p*< 0.05 if not otherwise stated.

For all analyses, we report the formula of the model using the Wilkinson notation in addition to the degrees of freedom, *t*- or *F*-values, significance of the respective regressors and their effect size in the **Supplementary Material**. A *post hoc* power analysis with an alpha-error of 0.05 was performed for each t-test and linear regression using the respective effect sizes and number of predictors (see **Supplemental Tables 1–4**). The power analysis was performed in G*Power [version 3.1; (63)].

## Results

Based on the role of *FTO* in DA signaling, its association with trait impulsivity (which is determined by DA function in midbrain neurocircuitry), and strong evidence for *FTO* affecting cell proliferation and neurogenesis suggesting a role for *FTO* in neurodevelopment, we tested whether hardwired, structural connectivity changes between *FTO* risk allele carriers and non-carriers in meso-striatal circuitry contribute to an impulsive phenotype. We specifically considered the projections from the DA midbrain (VTA/SN) to the ventral striatum and tested whether the impulsive phenotype observed in risk allele carriers was mediated by such structural changes. Additionally, we also measured structural connectivity between VTA/SN and the caudate, which is implicated in attentional impulsivity (64) but not primarily in motor impulsiveness, to verify the specificity of the expected effect of gene variant on connectivity between the midbrain and the nucleus accumbens. We also tested if the observed differences have an underlying sex dimorphism.

### Similar metabolic status and self-reported food intake behavior in risk allele carriers and non-carriers

In total, eighty-one participants were included in the final analysis (**Table 1**), 39 non-carriers (FTO^–^; 24.8 ± 3.5 years, 22.6 ± 2.6 kg/m^2^) and 42 risk allele carriers (FTO^+^; 25.7 ± 4.4 years; 23.2 ± 3.2 kg/m^2^), who were matched for age, BMI and sex. All participants self-reported to be healthy without any known diagnoses. Accordingly, participants in both groups did not differ in their metabolic status as measured by fasting insulin, glucose, cholesterol, and triglyceride values (all p > 0.4; **Table 1**). Neither risk allele carriers nor non-carriers reported depressive symptoms according to the BDI-2 (65) nor alcohol use disorder according to the alcohol use disorder identification test (66). Sensitivity to punishment and reward as well as eating behavior as assessed using the German version of the three-factor eating questionnaire were comparable across groups (all p > 0.05, **Table 1**).

### Greater motor impulsivity in *FTO* risk allele carriers than in non-carriers

**Figure 2:**
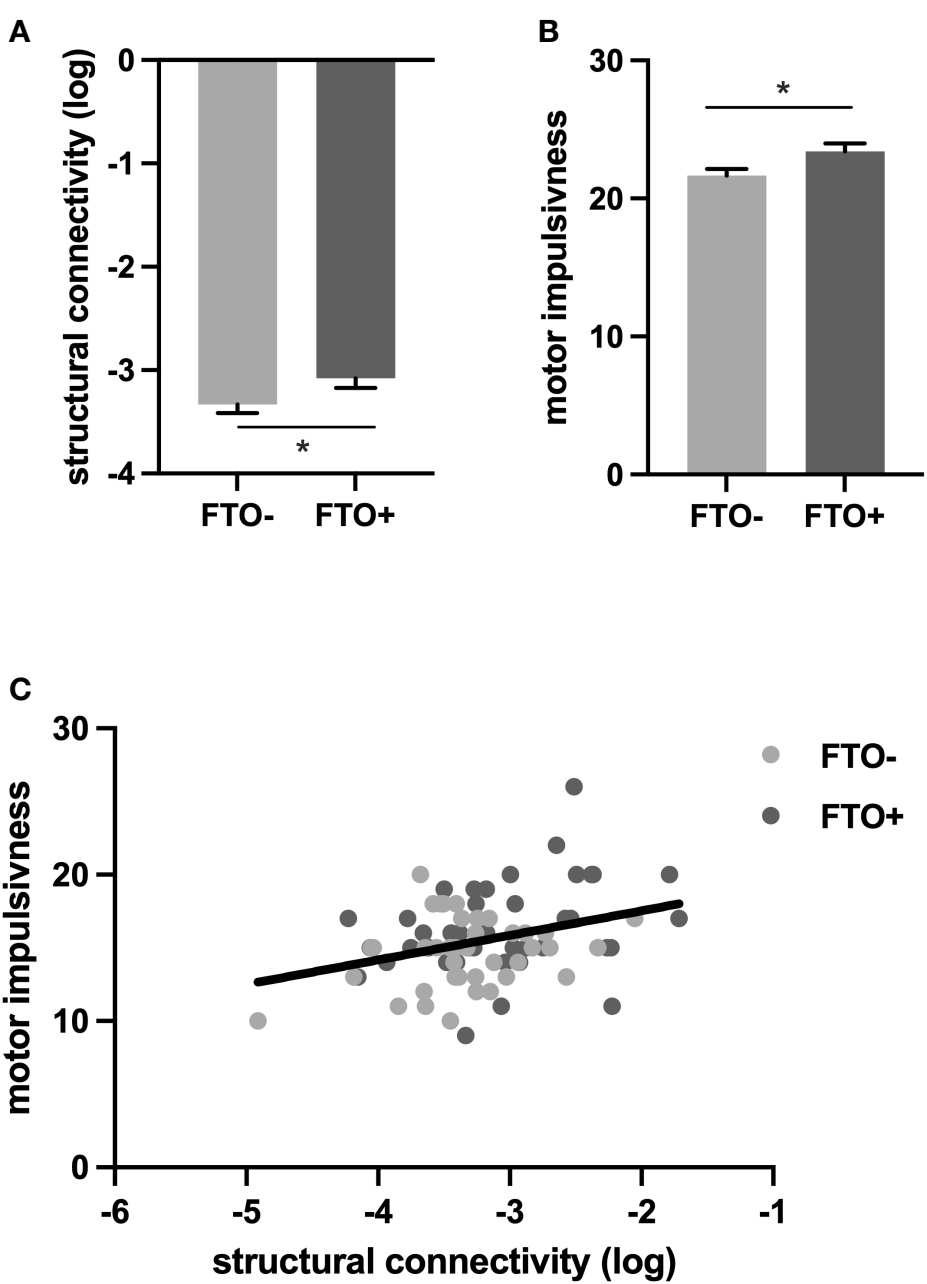
FTO allele status affects impulsivity and structural connectivity. **(A)**. Log structural connectivity is higher in the FTO+ group compared to the FTO–group. **(B)** Motor impulsiveness is higher in the FTO+ than in the FTO– group **(C)** Increased (log) structural connectivity is associated with increased motor impulsivity. *p < 0.05; rs9939609 T/A variant, FTO− group: TT; FTO+ group: AT, AA.

We first tested, whether *FTO* risk allele carriers differ in their impulsive phenotype in comparison to the non-carriers. Trait impulsivity in general, as measured by the BIS-11 total score, did not differ between the *FTO* ^+^ and the *FTO^–^* group (*F* = 2.08, p = 0.156, **Supplemental Table S1**). As motor and attentional impulsivity have been particularly associated with weight gain, with motor impulsiveness being strongly linked to DA functioning in the ventral striatum and attentional impulsiveness in the caudate (64), we next assessed if motor impulsiveness differed between the *FTO* ^+^ and *FTO* ^–^ groups. Compared to controls, *FTO* risk allele carriers demonstrated increased motor impulsiveness (*F =* 6.63, *p* = 0.030 after adjustment for multiple comparisons using Bonferroni correction), while no significant differences between *FTO* risk allele carriers and non-carriers could be observed for the other second-order factors of the BIS-11 including attentional impulsiveness (**Supplemental Table S2**, **Figure 2A**). Neither motor impulsivity nor the effect of FTO risk allele status on motor impulsivity differed between sexes (**Supplemental Tables S1, S2**).

### Increased structural connectivity between the midbrain and the nucleus accumbens in *FTO* risk allele carriers but not between the midbrain and the caudate nucleus in the same participants

We next analyzed, if structural connectivity is changed in *FTO* risk allele carriers compared to non-carriers. We particularly considered connectional changes between VTA/SN and NAc due to their relevance in encoding motor impulsiveness (40) and the link of *FTO* to DA functioning. Additionally, we also measured structural connectivity between VTA/SN and the caudate nucleus, which is implicated in attentional impulsivity (64) but not primarily in motor impulsiveness, to verify the specificity of the expected effect of gene variant on connectivity between the midbrain and the NAc.

We found that connectivity between the VTA/SN and the NAc was increased in *FTO* risk allele carriers compared to non-carriers (*F* = 4.06, *p* = 0.044, **Supplemental Table S3**, **Figure 2B**) while we did not find a significant difference in structural connectivity between VTA/SN and the caudate (*FTO* ^+^ vs *FTO* ^-^ group; *F* = 1.17, *p* = 0.283, **Supplemental Table S3**). Neither VTA/SN – NAc nor VTA/SN-caudate connectivity nor the effect of FTO risk allele carrier status on either connectivity measurement differed between sexes (**Supplemental Table S3**).

### Increased structural connectivity between the midbrain and the nucleus accumbens mediates the effect of *FTO* risk allele carrier status on heightened motor impulsiveness

To assess, whether increased connectivity in meso-striatal pathways in *FTO* risk allele carriers contributes to the greater motor impulsivity also observed in the *FTO* ^+^ group, we applied a mediation analysis. We demonstrate that increased structural connectivity between VTA/SN and NAc mediates the effect of allele status on motor impulsivity. Specifically, 21% of the *FTO* effect on motor impulsivity was mediated by the structural connectivity between the VTA/SN and NAc (**Supplemental Tables S4, S5**, **Figure 2C**), indicating a hardwired predisposition, that may well relate to neurodevelopmental alterations in *FTO* risk allele carriers and contribute to their impulsive phenotype.

## Discussion

The obesity-promoting allele of *FTO* is a common polymorphism with a minor allele frequency of 40–45% in European ancestry populations and has a relatively large effect on BMI [0.35 kg/m2 per allele; equivalent to 1 kg for a person who is 1.7 m tall; 2]. However, the mechanisms by which *FTO* risk allele carrier status causes weight gain and weight-gain-promoting behavior are not fully unraveled yet. We reveal, that increased impulsivity of *FTO* risk allele carriers is partially mediated by increased structural connectivity between the encoding brain regions —the DA midbrain and the ventral striatum - indicating that structural alterations in *FTO* risk allele carriers might contribute to their obesity-promoting behavioral phenotype.

Our result demonstrating greater impulsivity in *FTO* risk allele carriers is in line with previous studies reporting alterations in impulsivity for *FTO* polymorphism (32–34). Impulsivity has been conceptualized as both a trait being an enduring facet of the personality as well as a transient state relating to a reaction to specific conditions in an environment (67). Both of these aspects of impulsive behavior have been shown to relate to obesity (68–70). From the structural perspective, we focused on trait impulsivity, which is the main readout of the Barrattt Impulsivity Scale. In our data, *FTO* risk allele carrier status was specifically associated with greater motor impulsivity —the tendency to act without thinking. Motor impulsivity has been shown to interactively drive the risk of weight gain by increasing binge eating frequency and the preference for sweet snacks (71–73). In other words, if *FTO* risk allele status is associated with greater motor impulsivity, *FTO* risk allele carriers have a higher risk to engage in behavior that promotes body weight gain without immediately considering the health-adverse consequences.

Even though a number of studies have shown a clear link between *FTO* risk allele status and impulsivity, the direction of this effect is inconsistent with some reporting greater impulsivity, while others report reduced impulsivity in association with *FTO* variants (33, 74). In this context it is important to consider, that impulsivity is not a rigid construct. Hence the methodological constraints of quantifying such a complex concept limit comparability between studies (if different measures are used). In addition, the association of carrier status and impulsivity seems to depend on the developmental state: *FTO* risk allele carrier status was associated with lower impulsivity in childhood (74) and young adulthood (33), but the reduction of impulsivity associated with aging is lower in *FTO* variant carriers compared to non-carriers (32) indicating an ongoing effect of FTO on development possibly obscured by compensatory mechanisms during aging.

Our results further revealed increased structural connectivity between the midbrain and the ventral striatum in *FTO* risk allele carriers, for which the underlying mechanism is unclear. One potential mechanism could comprise the developmental effect of FTO on neuronal and oligodendrocyte plasticity. Structural connectivity measurements can be considered a tool for assessing the neurodevelopmental impact of genes on human phenotypes (75), as structural connectivity seems to be under stronger genetic control than functional connectivity (76). On a molecular level, this is evidenced by the central role of gene-expression gradients in guiding axons to their targets as the brain develops (77). Genetic effects are not uniformly distributed across the connectome, but the connectivity of some neural elements is under stronger genetic control than others (76). Our results indicate that *FTO* variants affect the neurodevelopment of the meso-striatal projections. We could not detect altered connectivity between the DA midbrain and the caudate, indicating a potential specificity of the neurodevelopmental effects of *FTO* polymorphisms. However, these findings need to be interpreted with care, as the indirect nature of structural connectivity estimates does not allow any insight into the underlying molecular effects of *FTO* variants on the development of DA projections. Recent evidence from animal work indicates that *FTO* polymorphisms affect the expression of axon guidance molecules of striatal projections (78) pointing toward one possible molecular mechanism. Other mechanisms might include alteration in neuroinflammation (79) stress sensitivity and expression of neurotrophic factors such as BDNF (13, 80).

We further show that the structural alterations between the midbrain and the ventral striatum partially mediate the impulsive phenotype of risk allele carriers. This finding extends previous reports of altered functional activation of the NAc during various behavioral tasks in risk allele carriers (28, 30, 34, 81) and assessments of functional coupling between VTA/SN and NAcc during reward learning (31). It denotes that *FTO* polymorphism exerts its effect on behavior most likely through alterations in neuroplasticity, ie. during connectional maturation causing a predisposition for certain behavioral traits in addition to altering DA functioning.

While the here investigated structural connectivity between the midbrain and ventral striatum is based on a strong molecular basis involving FTO in DA encoding of impulsivity in this circuitry (8), it needs to be kept in mind that impulsivity is a complex construct modulated and encoded by a multitude of brain circuitries, including the frontal cortex, for which a regulatory role of FTO cannot be precluded. Equally, while we neither find differences in attentional impulsivity nor in the structural connectivity of its encoding circuitry (the midbrain projections to the caudate), the genetic architecture of disease-associated FTO loci may involve extensive pleiotropy and shared allelic effects across tissues including temporally restricted effects. Indeed, the caudate has been established as one site of IRX3 expression, which is the long-range target of obesity-associated variants of FTO (26), rendering it a likely site-of-action of the obesity-associated FTO variants, for which the functional role has yet to be fully clarified and might extend to other behavioral facets than impulsivity.

In interpreting the results, it is important to consider, that we assessed structural connectivity and impulsivity in healthy-weight young adults ensuring that identified changes can be attributed to the *FTO* risk allele status and are not confounded by secondary effects of BMI, weight gain, or aging. This is particularly relevant because FTO expression is subject to nutritional regulation (82) indicating that environmental factors can interact with the genetic risk status and further complicate the association between allele status and related phenotype. As it is well established that obesity is a result of a Gene X environment interaction, investigating the mechanisms by which FTO polymorphisms drive body weight gain is critical to understand the pathophysiological underpinnings of overeating and establishing targeted treatment options. This is, in particular, relevant, since previous studies have demonstrated that a healthy lifestyle with a balanced diet and physical activity can attenuate the effect of FTO on obesity risk by approximately 30% (83–85). Moving forward, the use of genetic screenings can be considered to identify individuals at risk of gaining weight in an obesogenic environment and provide customized treatment options focusing on neurobehavioral adaptation and lifestyle-interventions.

### Limitations

While the selection of our participants ensures the attribution of the observed effects to *FTO* risk allele carrier status, inviting exclusively non-obese participants might at the same time introduce a sampling bias, by including a specific group of participants, who despite carrying risk alleles, are able to avoid weight gain. Second, even though we here aimed for conclusive evidence to support our molecularly well-grounded hypothesis, as in all candidate gene studies, the sample-sized dependent achieved power (0.73, **Table S4**) might represent a noteworthy limitation (86). Future particularly molecular studies will validate whether the proposed theories and concepts about the neurodevelopmental role of *FTO* as a contributor to obesity-promoting behavioral traits hold true in various scenarios. Moreover, probabilistic tractography only provides an indirect measure of structural connectivity and is not sufficient to detect alterations on the axonal level (87). It further needs to be considered that the here investigated polymorphism rs9939609 is in strong linkage disequilibrium with two further SNPs [rs8050136 and rs9930506] in Europeans, rendering it impossible to fully attribute the observed phenotype to the rs9939609 SNP (88, 89). Intriguingly, in individuals of African descent, results linking rs9939609 to increased BMI are more heterogenous so that further studies a required to disentangle the effect of FTO polymorphisms in different ethnicities (90, 91). These limitations and possibly differential effects of the various *FTO* polymorphisms on neurodevelopment might contribute to the inconsistent findings so far linking *FTO* polymorphism with either altered structural connectivity (92, 93) or no connectivity changes (94). With increasing data availability and computational power, future (genome wide) association studies will be required to replicate the demonstrated relationship between allelic variation and brain connectivity across a more heterogenous sample including further (cortical) brain circuitries involved in encoding of impulsivity.

Overall, our data reveals that increased motor impulsiveness in *FTO* risk allele carriers is partially mediated by enhanced structural connectivity between the midbrain and ventral striatum indicating that *FTO* polymorphism affects obesity promoting behavior via structural alterations in addition to complementary mechanisms. Further studies are needed to scrutinize the underlying molecular mechanisms.

## Data availability statement

The data reported in this study cannot be deposited in a public repository per GDPR and IRB data protection policies. To request access, please contact [Marc Tittgemeyer, Max Planck Institute for Metabolism Research, tittgemeyer@sf.mpg.de]. Data provision may include processed and unprocessed data and will require a data- sharing agreement.

## Ethics statement

The studies involving human participants were reviewed and approved by the local ethics comittee of the medical faculty of the University of Cologne (10-228).The participants provided their written informed consent to parcipate in this study.

## Author contributions

SET: Conceptualization, Software, Methodology, Formal analysis, Investigation, Writing; RH: Methodology, Software, Formal analysis, Validation, Writing; CM: Software; MT: Funding acquisition, Conceptualization, Supervision. All authors contributed to the article and approved the submitted version.
