# Supplementary material for: Increased meso-striatal connectivity mediates trait impulsivity in FTO variant carriers
Source: Front Endocrinol (Lausanne). 2023 May 8;14:1130203. doi: 10.3389/fendo.2023.1130203 (PMC10200952; doi:10.3389/fendo.2023.1130203)
Supplement: Supplementary file 1 [file Table_1.docx]

**Supplemental tables**

**Table S1: Difference of BIS-11 Total score between the FTO^+^ group and the FTO^-^ group**

BIS-11 total ~*FTO* risk allele carrier status*Sex

|  | ***Df*** | ***F*** | ***p-value*** | ***η^2^*** | **Achieved power** |  |
| --- | --- | --- | --- | --- | --- | --- |
| FTO status | 1 | 2.08 | 0.156 | 0.02 | 0.18 |  |
| Sex | 1 | 3.26 | 0.075 | 0.04 | 0.33 |  |
| FTO status : Sex | 1 | 0.18 | 0.669 | 2.20e-03 | 0.06 |  |

*Df = degrees of freedom, η*^2^ = eta squared

**Table S2: Difference of BIS-11 second-order factors between the FTO^+^ group and the FTO^-^ group**

BIS-11 second order factor ~*FTO* risk allele carrier status*Sex

|  | *Df* | *F* | *p-value** | *η^2^* | Achieved power |  |
| --- | --- | --- | --- | --- | --- | --- |
|  | **Attentional impulsiveness** | | | | | |
| FTO status | 1 | 0.03 | 1 | 3.75e-04 | 0.05 |  |
| Sex | 1 | 1.15 | 0.864 | 0.01 | 0.11 |  |
| FTO status :sex | 1 | 0.67 | 1 | 7.98e-03 | 0.10 |  |
|  | **Motor impulsiveness** | | | | | |
| FTO status | 1 | 6.63 | 0.030 | 0.07 | 0.54 | * |
| Sex | 1 | 2.39 | 0.378 | 0.03 | 0.26 |  |
| FTO status :sex | 1 | 1.26 | 0.798 | 0.01 | 0.11 |  |
|  | **Nonplanning Impulsiveness** | | | | | |
| FTO status | 1 | 0.81 | 1 | 9.67e-03 | 0.11 |  |
| Sex | 1 | 0.26 | 0.408 | 0.03 | 0.26 |  |
| FTO status :sex | 1 | 0.26 | 1 | 3.08e-03 | 0.07 |  |

*Df = degrees of freedom*

*****p-values adjusted for multiple comparisons using Bonferroni correction

**Table S3: Difference of structural connectivity between the FTO^+^ group and the FTO^-^ group**

Log (VTA/SN-NAc) ~ *FTO* risk allele carrier status *Sex

and

Log (VTA/SN-Caudate) ~ *FTO* risk allele carrier status * Sex

|  | ***regressor*** | ***Df*** | ***F*** | ***p-value*** | ***η^2^*** | **Achieved power** |  |
| --- | --- | --- | --- | --- | --- | --- | --- |
| **VTA/SN- NAc** | *FTO* risk allele carrier status | 1 | 4.06 | 0.044 | 0.05 | 0.41 | * |
|  | *Sex* | 1 | 0.002 | 0.969 | 1.88e-05 | 0.05 |  |
|  | *FTO* risk allele carrier status : Sex | 1 | 0.03 | 0.874 | 3.10e-04 | 0.05 |  |
| **VTA/SN – Caudate** | *FTO* risk allele carrier status | 1 | 1.17 | 0.283 | 0.01 | 0.11 |  |
|  | *Sex* | 1 | 2.73 | 0.102 | 0.03 | 0.26 |  |
|  | *FTO* risk allele carrier status : Sex | 1 | 0.59 | 0.445 | 7.14e-03 | 0.09 |  |

*Df = degrees of freedom, η*^2^ = eta squared , NAc = Nucleus accumbens, SN = Substantia nigra, VTA = Ventral tegmental area

**Table S4: Effect of FTO status and VTA/SN-NAc connectivity on motor impulsivity**

BIS motor ~ FTO status*Gender + log(SN/VTA-NAc)

| ***regressor*** | ***Df*** | ***F*** | ***p-value*** | ***η^2^*** | **Achieved power** |  |
| --- | --- | --- | --- | --- | --- | --- |
| FTO status | 1 | 5.84 | 0.018 | 0.07 | 0.54 | * |
| Sex |  | 2.58 | 0.112 | 0.03 | 0.26 |  |
| Log(SN/VTA-NAc) | 1 | 6.99 | 0.010 | 0.08 | 0.60 | * |
| FTO status : sex |  | 1.62 | 0.206 | 0.02 | 0.18 |  |

*Df = degrees of freedom, η*^2^ = eta squared

**Table S5: Mediation analysis *FTO* risk allele carrier status 🡪 VTA/SN-NAc connectivity 🡪 motor impulsivity**

Model mediator: Log (VTA/SN-NAc) ~ *FTO* risk allele carrier status

Model full: BIS motor ~ *FTO* risk allele carrier status + log (VTA/SN-NAc)

|  | Estimate | *95% CI lower* | *95% CI upper* | *p-value* |  |
| --- | --- | --- | --- | --- | --- |
| ACME | 0.36 | 0.00 | 0.95 | 0.044 | * |
| ADE | 1.28 | 0.01 | 2.52 | 0.042 | * |
| Total | 1.65 | 0.42 | 2.92 | 0.008 | ** |
| Prop. Mediated | 0.21 | 0.00 | 0.76 | 0.048 | * |

Note. ACME = average causal mediation effects*,* ADE *=* average direct effects, Total = total effect of FTO carrier status and connectivity on motor impulsiveness, Prop. Mediated = proportion mediated, CI = confidence interval, NAc = Nucleus accumbens, SN = Substantia nigra, VTA = Ventral tegmental area
